# The Different Outcomes of Electrochemical Copolymerisation: 3-Hexylthiophene with Indole, Carbazole or Fluorene

**DOI:** 10.3390/polym11020355

**Published:** 2019-02-18

**Authors:** Karolina Gebka, Tomasz Jarosz, Agnieszka Stolarczyk

**Affiliations:** 1 Department of Physical Chemistry and Technology of Polymers, Silesian University of Technology, 9 Strzody Street, 44-100 Gliwice, Poland; Karolina.Gebka@polsl.pl (K.G.); Tomasz.Jarosz@polsl.pl (T.J.); 2 Department of Inorganic Chemistry, Analytical Chemistry and Electrochemistry, Silesian University of Technology, 6 Krzywoustego Street, 44-100 Gliwice, Poland

**Keywords:** poly(3-hexylthiophene), electrochemical copolymerisation, indole, carbazole, fluorene

## Abstract

Electrochemical polymerisation is reported to be a method for readily producing copolymers of various conjugated molecules. We employed this method for mixtures of indole, carbazole or fluorene with 3-hexylthiophene (HT), in order to obtain their soluble copolymers. Although polymer films were obtained, infrared (IR) and Raman investigations showed that instead of the expected linear copolymers, polyindole and polycarbazole *N*-substituted with HT, as well as a poly(3-hexylthiophene) (PHT)/polyfluorene blend were produced instead. Boron trifluoride diethyl etherate was also used in an attempt to promote copolymerisation, but the produced deposits were found to be highly degraded.

## 1. Introduction

Materials based on conjugated polymers have found wide commercial application, particularly in the development of electrochromic devices (ECDs) [1,2], photovoltaic (PV) cells [3] and organic field effect transistors (OFETs) [4] due to their beneficial electronic and mechanical properties [5,6,7,8,9,10]. The quest of fabricating ever newer generations of optoelectronic devices, however, inherently leads to greater requirements in terms of material properties. This in turn fuels the search for new systems, well-evidenced by the number of reports concerning novel conjugated moieties. Among the numerous highlights of such an approach, there is but one drawback, namely the synthetic effort required for the preparation of increasingly convoluted systems. Therefore, many valiant research efforts have been aimed at exploring an alternative approach—developing new qualities based on existing, well-investigated systems. One aspect of this approach is to blend the qualities of two or more conjugated units within a single layer, giving rise to polymer blends and copolymers [10,11,12,13,14,15,16]. Interestingly, the fruits of such labours may sometimes exceed expectations, exhibiting properties superior to those of the original systems [17,18].

Numerous routes to preparation of conjugated copolymers may be discerned, depending on the desired architecture of the copolymer—graft copolymers [19,20,21,22,23], alternating copolymers [24,25], multi-block [26,27] and statistical [23,28] copolymers, have all been reported. Among those systems, macromolecules consisting only of conjugated units [15,29,30], as well as those consisting of both conjugated and non-conjugated units [18,31,32] have been prepared and investigated. The synthesis and purification of such species is, however, often both time- and cost-intensive, particularly when well-defined systems, tailored for a particular application are desired [15,32].

The search for new materials, unlike the aforementioned optimisation of an established material, often requires the investigation of the properties of hundreds if not thousands of co-monomer combinations. As such, the best approaches to this process are both time- and cost-efficient. The synthesis of materials for this kind of initial evaluation typically relies on either of two approaches: fabrication of a co-oligomer, incorporating several linked systems and its subsequent polymerisation or direct copolymerisation between two distinct co-monomers. The former may be quite demanding in terms of synthetic procedures, while the latter might yield a mixture of homopolymers rather than the desired copolymer.

Electrochemical (co)polymerisation methods have found wide application as a cost-efficient solution in both of the above approaches, employing electrical stimuli instead of standard chemical reagents [33,34,35]. Although the procedural simplicity of electrocopolymerisation makes it an attractive technique, it is not without limitations. These constraints are related to the compatibility of co-monomers, as the behaviour of different conjugated systems during electrochemical polymerisation varies significantly. The prime factor being reported to determine whether two compounds undergo electrochemical copolymerisation [36,37], is the difference in their oxidation potentials—the larger the voltage “gap”, the less likely a co-monomer system is to yield a copolymer. Another factor that influences the electrocopolymerisation process is the ratio of molecular weights of the co-monomers, as reported in the ground-breaking work by Fuchigami et al. [38,39].

Although these two factors are known to influence copolymerisation, the magnitude of their impact on the process has not yet been investigated. Striving to compare these two effects, a co-monomer system should be selected so that they exert opposing influences over the process. In line with this assumption, the co-monomer possessing lower molecular weight should undergo oxidation at more positive potentials than the co-monomer with higher molecular weight.

We have often worked with 3-hexylthiophene (HT) and its polymers. Seeing their good solubility in organic solvents, as well as the favourable electronic properties of poly(3-hexylthiophene) (PHT), in our search for new materials, we opted to maintain 3-hexylthiophene as one of the co-monomers and investigate the possibility of coupling it with other conjugated co-monomers. Of those conjugated co-monomers, polycarbazole (Cz), as well as its numerous derivatives are well-known materials for organic electronics, particularly due to their thermal and photochemical stability, high triplet energy and favourable hole-transporting properties [40,41]. Indole (In) itself is relatively less-known, but nevertheless a material showing several beneficial properties good thermal stability, high-redox activity and stability, and slow degradation rate in comparison with polyaniline and polypyrrole [33,42,43,44] that could favourably supplement the properties of PHT, if successfully copolymerised.

Considering the message conveyed by the recent work on identifying copolymers by Holze [45], we saw the merit in employing extensive infrared (IR) and Raman spectroscopic measurements as means for identifying the structural details of the resultant polymeric layers. The need for the use of these methods is particularly relevant, in light of the results obtained.

## 2. Materials and Methods

### 2.1. Materials

**Scheme 1:**
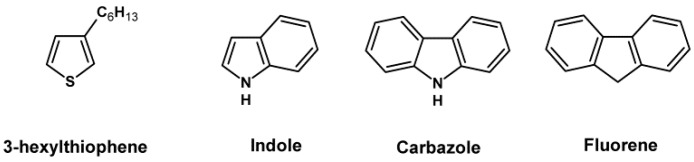
Chemical formulae of the co-monomers investigated.

3-hexylthiophene (HT) ≥ 98%, TCI (Tokyo, Japan), indole (In) > 99%, carbazole (Cz) > 95%, fluorene (Fl) > 98% (Scheme 1), chloroform > 99.5% and boron trifluoride diethyl etherate > 46.5% BF_3_, Sigma Aldrich (Saint Louis, MO, USA) were used as received.

### 2.2. Electrochemical Polymerisation and Spectroelectroconductometric Meausrements

Solutions for electrochemical and spectroelectrochemical investigations were prepared by dissolving the co-monomers in acetonitrile (Sigma-Aldrich, Saint Louis, MO, USA, CHROMASOLV, >99.9%, HPLC grade). The concentrations of co-monomers in the investigated solutions are given in Table 1.

Electrochemical (co-)polymerisation was performed using a standard cell, with an Ag pseudo-reference electrode and a Pt coil counter electrode. Three types of working electrodes were used, depending on the follow-up investigations:

- Standard cyclic voltammetry measurements—we used glass plates, on whose surface two Pt working electrodes were deposited in an interdigitated array configuration. The interdigitated section consisted of 500 whisker pairs, with a Pt path width of 5 μm and path spacing of 5 μm (Dropsens, Oviedo, Spain);
- IR spectroscopic investigations—Pt flag working electrodes were used, as they were able to deform without breaking when pressed to the surface of the attenuated total reflection (ATR) crystal of the IR spectrometer;
- Ultraviolet-visible-near infrared (UV-Vis-NIR) spectroscopic measurements—we used thin glass slides (90 mm × 7 mm × 0.3 mm), on whose surface two Pt working electrodes were deposited in an interdigitated array configuration. The design of the interdigitated section was identical to the one detailed above; the electrodes were custom-produced by Micrux (Oviedo, Spain).

Electrochemical data were acquired using a Metrohm-Autolab PGSTAT302N (Herisau, Switzerland) potentiostat.

UV-Vis-NIR spectra were recorded using an Ocean Optics (Largo, FL, USA) diode-array spectrometers set (QE65000 and NIRQuest 512).

A solution of 0.1 M tetrabutylammonium tetrafluoroborate, Bu_4_N^+^BF_4_^−^ (Sigma-Aldrich, >99.0%, electrochemical analysis grade) in acetonitrile was used in all experiments as the supporting electrolyte. Prior to measurement, each sample investigated was purged with inert gas, while the same gas was being passed through the electrochemical cell during the measurement. After any polymerisation experiment, the working electrode was thoroughly rinsed with acetonitrile to remove any monomer and electrolyte residue.

Applied potentials in all of the electrochemical experiments were calibrated versus the ferrocene/ferrocinium redox couple as presented.

IR spectroscopy was carried out on a Perkin-Elmer Spectrum Two (Waltham, MA, USA) spectrometer, equipped with an universal attenuated total reflectance (UATR) (Single Reflection Diamond) module.

## 3. Results and Discussion

### 3.1. Electrochemical Polymerisation and Cyclic Voltammetry of Layers Obtained

**Figure 1:**
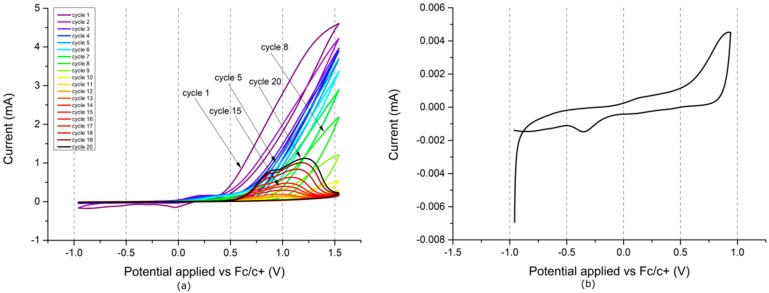
Electrochemical polymerisation of indole (**a**), CV of the deposited layer (**b**).

Electrochemical polymerisation of indole was carried out at a potential range from −0.95 to +1.54 V (Figure 1a). The first cycle shows a quasi-reversible redox pair, with the oxidation peak at approx. +0.27 V and the reduction peak at approx. −0.03 V. Such a redox pair has also been reported for indole, although it is unclear to what transition it corresponds [46]. A sharp oxidative slope is seen with an onset at approx. +0.4 V. This slope gradually decreases and shifts towards higher potentials with subsequent polymerisation cycles, indicating low conductivity of the deposited film. As the polymer film grows, a new oxidation signal begins evolving between +0.6 and +1.1 V, transforming into two oxidation peaks, centred at +0.88 V and at +1.22 V, by the 20th polymerisation cycle. Interestingly, this signal is not accompanied by any noticeable reduction signals. This may indicate that no significant doping (and associated dedoping) of the polymer takes place during potential cycling, which would imply that the oxidation signals are those of polymer over-oxidation or charging, yielding localised charged defects rather than delocalised charge carriers.

The cyclic voltammetric (CV) curves of the polymer layer recorded at 0.001 V/s (Figure 1b) show traces of the low potential redox pair, with a small oxidation signal at +0.16 V and an even less pronounced reduction signal at +0.05 V. An oxidation signal is present, with an onset at approx. +0.41 V and predicted maximum at +0.92 V. Interestingly, at this low potential scanning rate, a well-pronounced reduction peak is observed, centred at −0.34 V. The occurrence of this signal, as opposed to its lack at higher potential scanning rates (e.g., during polymerisation) may indicate that the reduction process is severely limited, possibly due to issues with conjugation in the polyindole layer.

**Figure 2:**
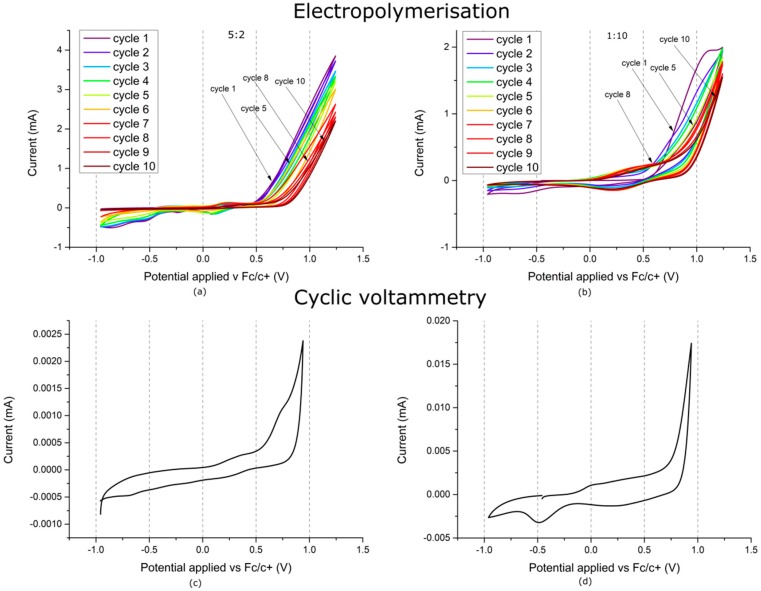
Electrochemical polymerisation of In-HT 5:2 (**a**), In-HT 1:10 (**b**), CVs of deposited In-HT 5:2 EP (**c**), In-HT 1:10 EP (**d**) layers.

When electrochemical polymerisation is performed for In-HT 5:2 (Figure 2a), the low potential redox pair observed for indole is also present (oxidation at +0.22 V and reduction at +0.08 V), as well as an oxidative slope, with an onset at approx. +0.7 V. An oxidative plateau spanning the range from −0.33 to +0.03 V is an additional feature, evolving during film deposition. In the cathodic half-cycle, reduction signals are observed at −0.56 V and at −0.86 V, but diminish gradually with subsequent polymerisation cycles. Similar to the polymerisation of indole, the oxidative slope steadily shifting towards more positive potentials, but no high-potential oxidation signals are formed.

In the case of electrochemical polymerisation conducted for In-HT 1:10 (Figure 2b), a large oxidation signal, with a maximum at +1.14 V, followed by a small oxidative slope at higher potentials is observed. In the subsequent potential cycles, the signal declines sharply and evolves into an oxidative slope. Simultaneously, a redox pair begins developing, with an oxidation signal centred at approx. +0.4 V and a reduction peak centred at +0.23 V. 

**Figure A1:**
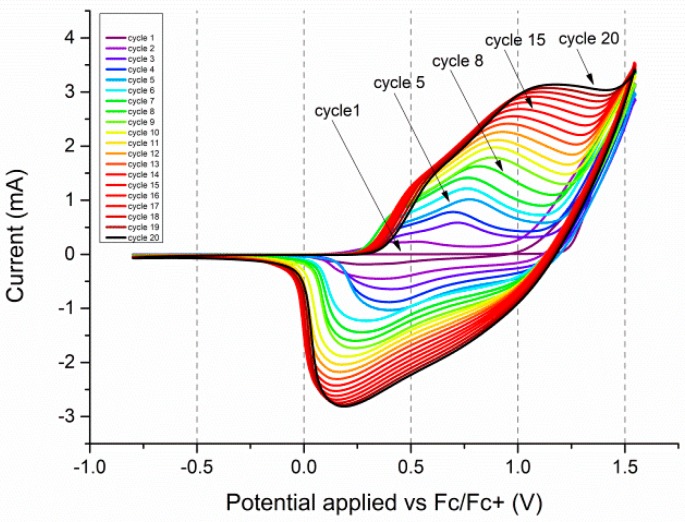
CV recorded during the electrochemical polymerisation of 20 mM HT in 0.1 M tetrabutylammonium tetrafluoroborate.

The CV of the layer obtained via polymerisation of In-HT 5:2 (Figure 2c) shows a small oxidation signal at +0.73 V, followed by an oxidative slope at higher potentials, with the latter possibly corresponding to over-oxidation of the film. Unlike polyindole, this film does not show any pronounced reduction signals in the cathodic half-cycle. This is not the case for the layer prepared from In-HT 1:10 (Figure 2d), as a reduction signal is observed at −0.48 V. This film also shows traces of an oxidation signal at 0 V, most likely being the low-potential redox pair seen for polyindole. Interestingly, neither CV shows any features typical for oligo(HT) or PHT (Figure A1), indicating that if HT units are present in the layer, they do not form any longer segments, possibly being limited to only monomeric or dimeric species.

**Figure 3:**
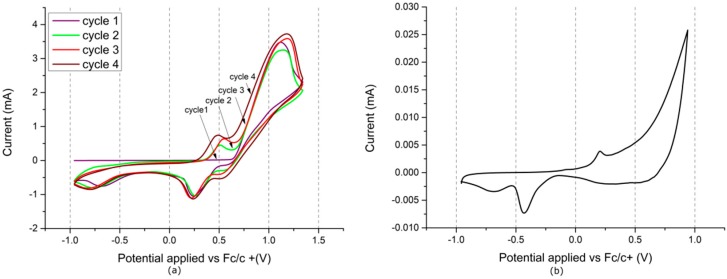
Electrochemical polymerisation of carbazole (**a**), CV of the deposited layer (**b**).

During electrochemical polymerisation of carbazole (Figure 3a) in the range from −0.96 to +1.34 V, a strong oxidation signal is observed at +1.18 V in the anodic half-cycle and a reduction shoulder typical for carbazoles is seen at approx. +0.55 V. In the following cycles, a second oxidation peak, at +0.54 V evolves, along with a corresponding reduction signal at +0.22 V. This pair of signals corresponds to the doping and dedoping of the deposited polymer film, showing its high electroactivity.

The CV of the layer obtained during polymerisation (Figure 3b) shows a small oxidative peak at +0.21 V and an oxidative slope with an onset at approx. +0.46 V. It also shows a broad reduction signal, centred around approx. +0.4 V and a sharp reduction peak at −0.43 V. These are likely due to the low employed potential scanning speed, resulting in the broadening of the doping/dedoping signals, with the +0.54 V oxidative peak seen during polymerisation forming the oxidative slope and the reduction peak and reduction shoulder merging and forming the extremely broad reduction signal at +0.4 V. The two small signals (oxidation +0.21 V and reduction at −0.43 V) are likely due to charge trapping and detrapping, only becoming noticeable during extremely slow potential scanning. 

**Figure 4:**
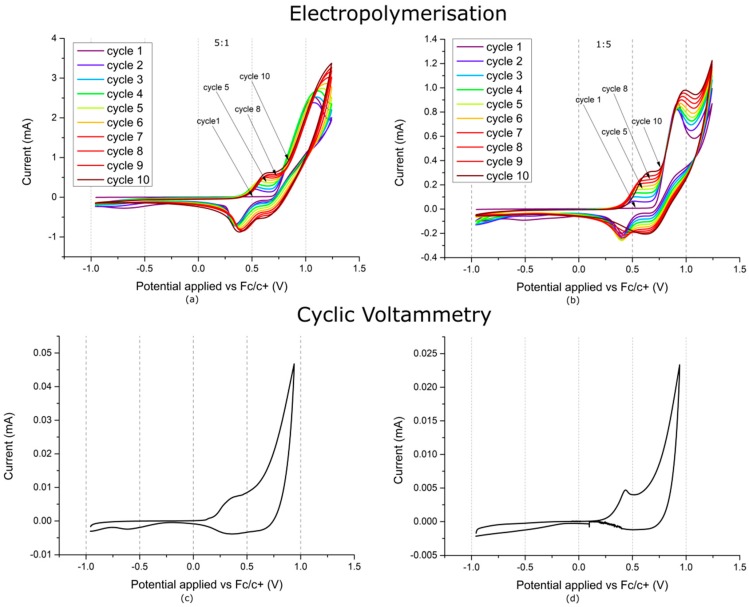
Electrochemical polymerisation of Cz-HT 5:1 (**a**), Cz-HT 1:5 (**b**), CVs of deposited Cz-HT 5:1 EP (**c**), Cz-HT 1:5 EP (**d**) layers.

The CV recorded during electrochemical polymerisation of Cz-HT 5:1 (Figure 4a) is similar to that for carbazole, but shows a more pronounced increase of the signals attributed to the doping and dedoping of the polymer.

When the ratios of the co-monomers are reversed (Figure 4b), the shape of the CV changes significantly. In the anodic half-cycle, a strong oxidation peak, typical for HT, is seen at +0.9 V. The cathodic half-cycle response is similar to that of carbazole, showing a reduction shoulder at +0.6 V and a subsequent reduction peak at +0.39 V. In the following potential cycles, oxidation and reduction signals begin evolving, respectively at +0.55 V and at +0.65 V. Unlike the polymerisation of carbazole, the oxidation signal takes the form of a shoulder rather than the peak seen for carbazole.

The CV of the layer prepared via electrochemical polymerisation of Cz-HT 5:1 (Figure 4c) shows a small oxidation peak at +0.34 V and an oxidative slope with an onset at approx. +0.55 V as well as a broad reduction peak at +0.70 V. In the case of layer prepared from Cz-HT 1:5 (Figure 4d), the oxidation peak is more pronounced and found at a slightly higher potential of +0.43 V. 

**Figure 5:**
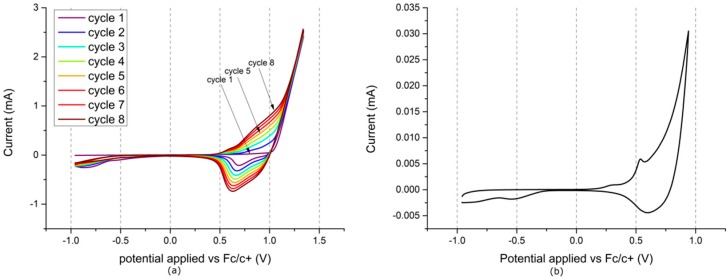
Electrochemical polymerisation of fluorene (**a**), CV of the deposited layer (**b**).

Electrochemical polymerisation of fluorene was carried out at potentials from −0.96 to +1.34 V (Figure 5a). The first cycle contains a sharp oxidative slope, with an onset at approx. 1.0 V, associated with the oxidation of the monomer. In the cathodic half-cycle, a reduction signal is found at +0.68 V, attributed to the de-doping of the polymer deposited in the anodic half-cycle. In subsequent cycles an oxidation signal evolves at approx. +0.8 V, associated with the doping of the deposited polymer film, steadily increasing along with the aforementioned reduction signal.

The CV of the layer obtained during polymerisation (Figure 5b) shows a small oxidative peak at +0.54 V, superposed onto an oxidative slope that is similar in shape to the polymer film doping signal, and a broad reduction signal, centred at approx. +0.6 V.

**Figure 6:**
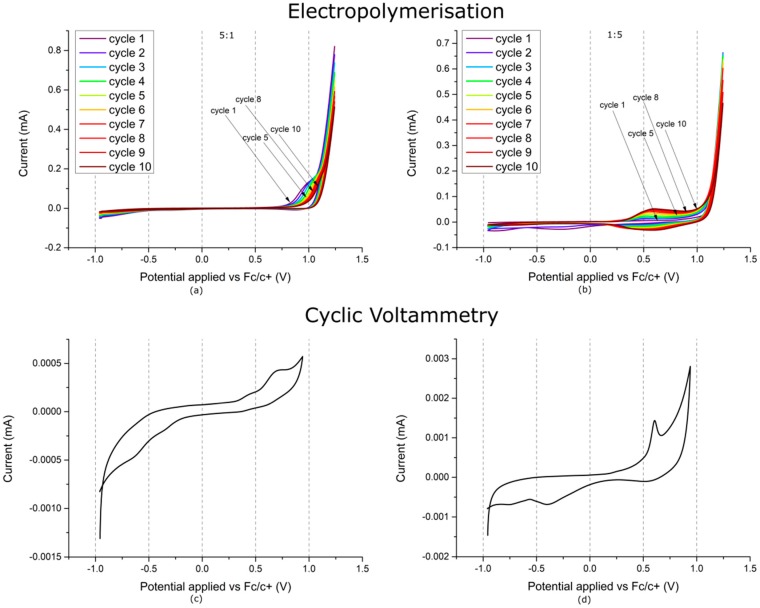
Electrochemical polymerisation of Fl-HT 5:1 (**a**), Fl-HT 1:5 (**b**), CVs of deposited Fl-HT 5:1 EP (**c**), Fl-HT 1:5 EP (**d**) layers.

Although the CV recorded during electrochemical polymerisation of Fl-HT 5:1 (Figure 6a) shows some similarity to that recorded for fluorene, curiously only very minor reduction signals are observed at +0.87 V and at approx. −0.3 V, both decreasing in subsequent cycles.

In the case of electrochemical polymerisation of Fl-HT 1:5 (Figure 6b), initially a faint oxidation signal is seen at +0.58 V prior to the oxidative slope associated with monomer oxidation. In the cathodic half-cycle, two reduction signals are observed, at −0.26 V and at −0.88 V, but are not seen in subsequent cycles, likely arising due to the “cloud” of oligomers, produced in the anodic half-cycle, reaching the counter electrode. In subsequent cycles, an oxidative shoulder develops, showing a faint maximum at +0.52 V and stretching towards higher potentials. This shoulder gradually develops into an oxidation peak +0.59 V. Simultaneously, a reduction signal evolves into a peak at +0.54 V. The two peaks arise due to the doping and dedoping of the deposited polymer film respectively. It is worth noting that, unlike the polymerisation of fluorene (Figure 5a) and that of the Fl-HT 5:1 (Figure 6a) mixture, the polymer doping and dedoping signals are quite pronounced and almost completely separated from the monomer oxidation signal, likely an effect of the large concentration of HT, which improves conjugation in the deposited polymer film.

The CV of the layer prepared via electrochemical polymerisation of Fl-HT 5:1 (Figure 6c) features a single oxidative signal at +0.70 V and two poorly-pronounced reduction signals at approx. −0.4 and −0.6 V. In the case of layer prepared from Fl-HT 1:5 (Figure 6d), the oxidation signal is located at slightly lower potentials and is well-pronounced, manifesting as a sharp peak at +0.60 V. Similarly to the polymer film prepared from the other co-monomer ratio, two reduction signals are seen at approx. −0.4 and −0.6 V, as well as a very broad reduction signal at +0.62 V, associated with the dedoping of the polymer film, more typically seen for PHT than for polyfluorene, possibly indicating a significant share of HT units being present in the polymer film.

### 3.2. Material Identification

**Figure 7:**
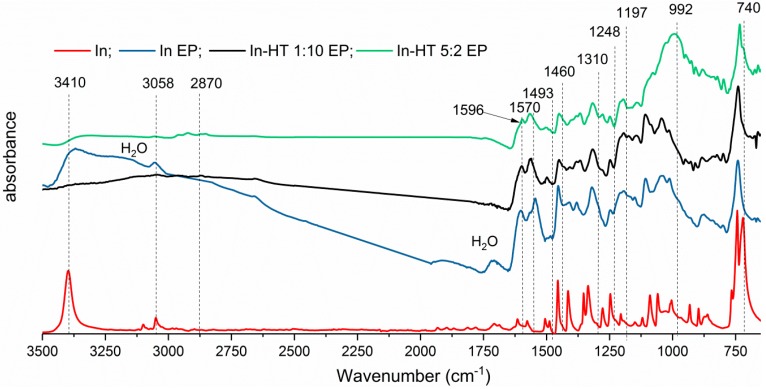
Attenuated total reflection-infrared (ATR-IR) spectra of indole, its homopolymer (In EP) layer and In-HT EP layers.

**Figure 8:**
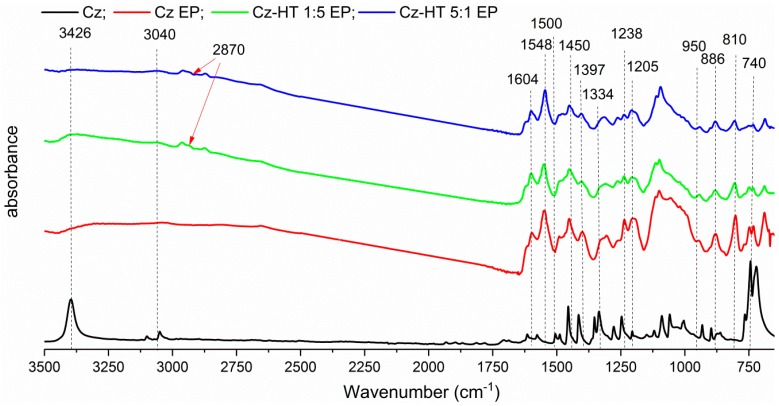
ATR-IR spectra of carbazole, its homopolymer (Cz EP) layer and Cz-HT EP layers.

**Figure 9:**
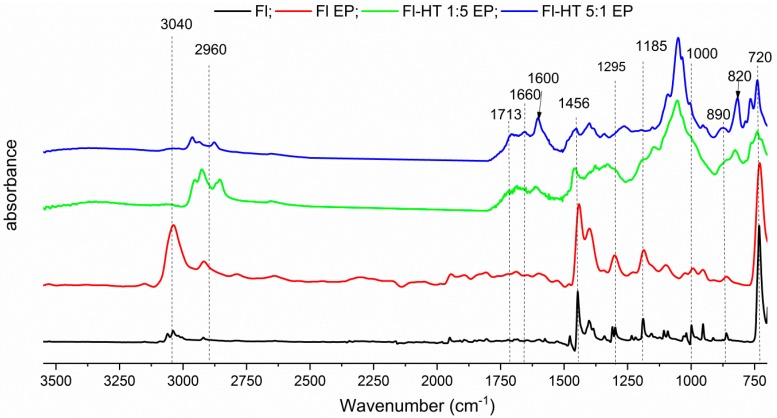
ATR-IR spectra of the fluorene, its homopolymer (Fl EP) layer and Fl-HT EP layers.

Structures of obtained materials were confirmed by comparative analysis of IR-ATR spectra of reagents and final products. The structure of the solid films obtained was identified using IR spectroscopy. The spectra recorded for both monomers and the products of their electrochemical polymerisation are presented in Figure 7, Figure 8 and Figure 9.

#### 3.2.1. Polyindole

For the In EP spectrum (Figure 7, Table A1), the strong peak at 740 cm^−1^ corresponds to out of plane deformation of C–H bond of the benzene ring in the indole moiety. Peaks at 1460 and 1493 cm^−1^, assigned to stretching modes of benzene rings, indicate that the benzene ring was not the polymerisation site. The absence of the band at 720 cm^−1^ confirms that the benzene ring was virtually unaffected during polymerisation. Instead, the C2 and C3 of the pyrrole ring were the sites, at which other indole repeat units were attached during polymerisation. The presence of both a signal at 3400 cm^−1^ and a peak at 1570 cm^−1^, attributed to N–H stretching and out of plane oscillations respectively, excludes the possibility of substitution (polymerisation) taking place at the indole nitrogen atom.

#### 3.2.2. Polycarbazole

The peak at 3426 cm^−1^ is the characteristic oscillation of the N–H bond, which broadens and shifts to 3418 cm^−1^ in the polycarbazole (PCz) spectrum (Figure 8, Table A1) and is extinguished for the product of electrochemical polymerisation of carbazole with 3-hexylthiophene (HT). In the range of 1600–1450 cm^−1^, C–C and C–N stretching oscillations of PCz are found. C–H oscillations in the range from 810 cm^−1^ to 740 cm^−1^ are a specific feature of a trisubstituted benzene ring, which indicates that polymerisation may take place via C3 and C6. Simultaneously, two signals at 1238 cm^−1^ and at 1205 cm^−1^, attributed to out of plane C–C oscillations, merge into a single band at 1237 cm^−1^, which unequivocally shows that a C–C bond was formed between the monomer molecules. Moreover, the bands at 1604 cm^−1^ and at 1458 cm^−1^, attributed to C=C and C–C stretching and shrinking oscillations, are broadened in relation to the ones observed on the spectrum of carbazole. This also indicates that the main component of the films obtained shows typical features of conjugated polymers, supporting the claim that the obtained product is PCz.

#### 3.2.3. Polyfluorene

In the IR spectra of Fl EP and Fl-HT-EP (Figure 9, Table A1), the modes at 3040 and 1450 cm^−1^ are assigned to aromatic C−H stretching. The peaks at 820 and 890 cm^−1^ are assigned to the C–H out of plane vibration of the benzene ring and the peak at 1000 cm^−1^ is due to the C–H in-plane vibration of the benzene ring. Furthermore, in the higher wavenumber region, the overtone (1750 cm^−1^) and the combination tone (1713 cm^−1^) of these vibrations were observed. These peaks are similar to those of 1,2,4-trisubstituted benzene derivatives, with respect to the wavenumber and the pattern of the absorption peaks. Therefore, the result suggests that the fluorene rings are linked either through the 2,7- or the 3,6-carbons.

#### 3.2.4. Discussion of Infrared (IR) and Raman Spectroscopic Results

**Scheme 2:**
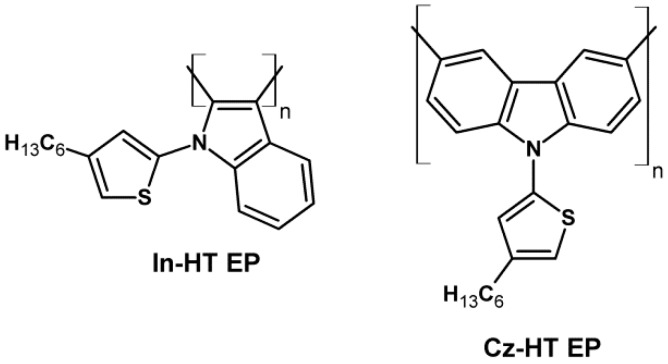
Proposed structural formulae for In-HT EP and for Cz-HT EP.

When indole and carbazole are polymerised in the presence of HT, the signals associated with the N−H bond are either largely diminished or completely extinguished. This feature can indicate that HT units are included in the polymer structure, substituting the NH hydrogen atom of both indole and carbazole polymers. Simultaneously, HT alkyl group signals are observed in these spectra at 2800−2900 cm^−1^ and thiophene C−C bond signals are also found at 1500 cm^−1^, confirming the presence of HT units in the polymer films obtained. Solubility testing of the supposed In-HT and Cz-HT copolymers showed that no chloroform-soluble products were extracted, supporting our claim of a permanent binding of HT to In and Cz through their respective aromatic nitrogen atoms (Scheme 2).

**Figure A2:**
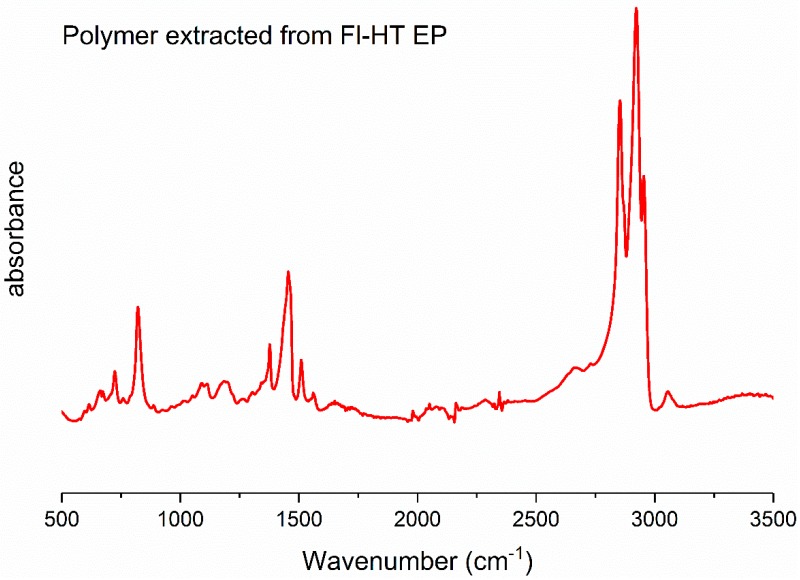
IR spectrum of the polymer fraction of Fl-HT EP soluble in chloroform and deposited from solution on the IR spectrometer ATR crystal.

In the case polymerisation of Fl with HT, all characteristic bands for PHT are located at 2960, 2925, and 2855 cm^−1^, which are attributed to aliphatic C−H stretching of PHT, as well as the 820 cm^−1^ band, corresponding to aromatic C−H bending. The signal at 1456 cm^−1^ is due to the bending vibration of the C−H bond, which could suggest that a block copolymer was obtained. During solubility testing, using chloroform, a polymer was isolated, yielding an orange chloroform solution and leaving a blackish residue on the electrode. The IR spectrum of the dissolved polymer clearly indicates that it is PHT (Figure A2). The fact that PHT was dissolved and no carbazole-originating signals are seen in the IR spectrum, clearly indicate that, at best, a blend of homopolymers was obtained during electrochemical polymerisation. 

**Figure A3:**
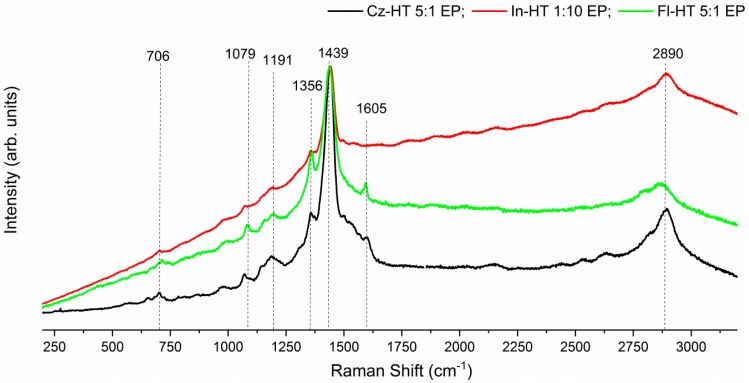
Raman spectra of the polymer films obtained during electrochemical polymerisation of In-HT, Cz-HT and Fl-HT.

Raman spectroscopic (Figure A3) measurements were conducted for all co-monomer mixtures. The Raman spectra for all copolymers are dominated by thiophene signals. The strongest band, observed at 1446 cm^−1^, is assigned to the in-phase C=C stretching vibration of the thiophene ring, whereas the band at 1382 cm^−1^ is assigned to the in-phase C−C stretching vibration of the thiophene ring. In the case of the Fl-HT EP layer, signals from the phenyl rings are hardly visible.

On this basis and that of an examination of characteristic mode frequencies, the peak at 1605 cm^−1^ to a symmetric substitution is assigned to the ring-stretching mode. The region of the spectrum between 1400 and 1000 cm^−1^ shows several peaks (1356, 1191, 1079, and 100 cm^−1^) that are weaker in intensity than those at 1605 cm^−1^, these peaks are mainly due to C–C stretching modes between phenylene rings.

In light of these results, we propose the following structural formulae for In-HT EP and for Cz-HT EP (Scheme 2):

### 3.3. Ultraviolet-Visible-Near Infrared (UV-Vis-NIR) Spectra of the Produced Films

**Figure 10:**
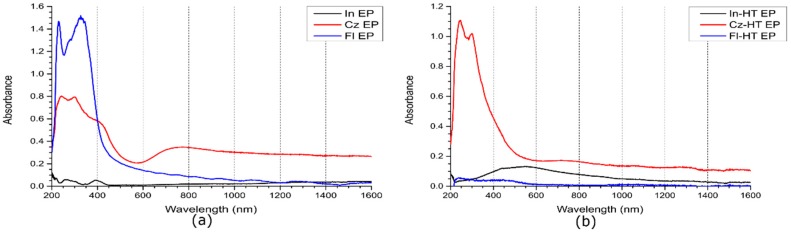
Ultraviolet-visible-near infrared (UV-Vis) spectra of polymeric films prepared from pure monomer solutions (**a**) and monomer solutions containing HT (**b**).

The UV-Vis spectra of the homopolymer films (Figure 10a) are in line with literature reports. In the case of both polycarbazole and polyfluorene, strong absorption signals are observed in the UV range (signals at 231 and 330 nm for polyfluorene and at 241 and 306 nm for polycarbazole) and Vis ranges (low-energy absorption shoulder starting at approx. 440 nm for polyfluorene and signals at approx. 409 nm and at 769 nm for polycarbazole). In the case of polyindole, only minor signals (several signals near 270 nm and a non-Gaussian signal at 393 nm) are observed, corresponding to the relatively small electrochemical response (Figure 1c,d) of the homopolymer.

Interestingly, despite employing similar conditions for the preparation of films from mixed co-monomer solutions (Figure 10b), only thin films were obtained for Fl-HT EP, while for both In-HT EP and Cz-HT EP, films thicker than those of the homopolymers were produced. In all three cases, increased absorption in the 400–600 nm range was observed, which is caused by the presence of readily conjugating HT units in the films obtained. Of the three types of such films, In-HT EP films showed absorption reaching towards the lowest energy, which may point towards the existence of charge carriers on short, oligo (HT) chains as the alternative; the formation of PHT chains would have been detected by IR spectroscopy beforehand. 

### 3.4. Copolymerisation in Boron Trifluoride Diethyl Etherate

In light of the obtained results, showing that instead of the expected linear copolymers, either “thiophene-grafted” homopolymers or homopolymer blends were obtained, we have attempted to induce copolymerisation by supplementing the experimental solutions with boron trifluoride diethyl etherate (10 vol %), as reported to be fruitful in literature for 3-methylthiophene [42,47,48].

**Figure A4:**
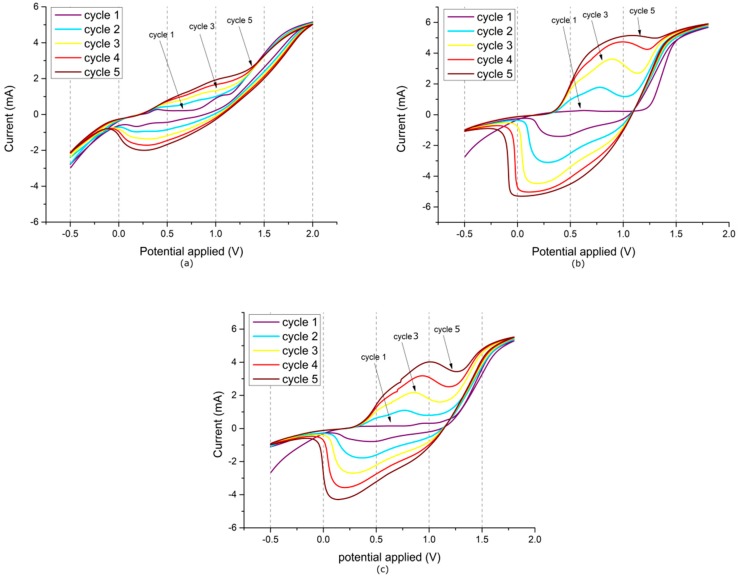
Electrochemical polymerisation of In-HT (**a**), Cz-HT (**b**), Fl-HT (**c**) containing 10 vol % boron trifluoride diethyl etherate.

**Figure A5:**
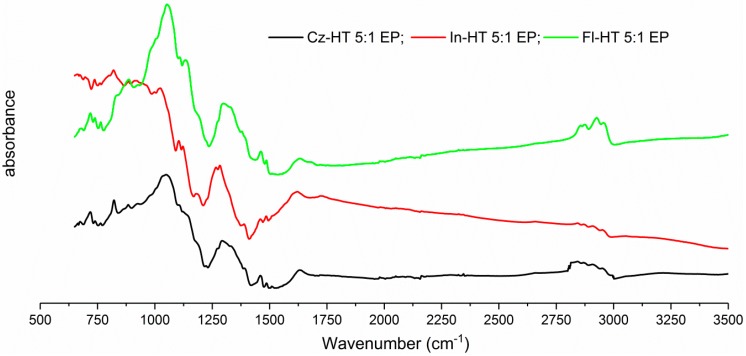
IR spectra of the polymer deposits obtained during electrochemical polymerisation of In-HT, Cz-HT and Fl-HT in the presence of 10 vol % boron trifluoride diethyl etherate.

**Figure A6:**
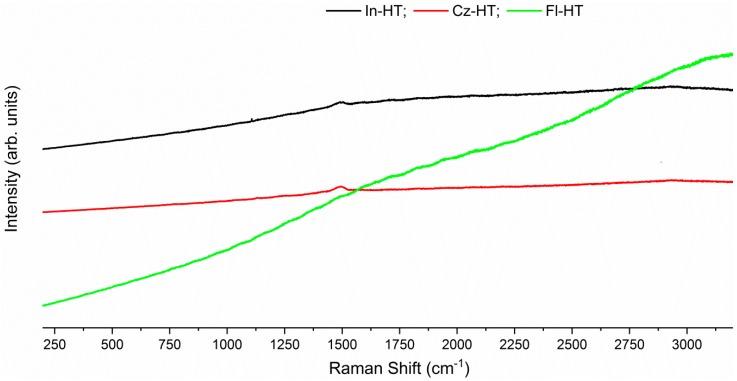
Raman spectra of the polymer deposits obtained during electrochemical polymerisation of In-HT, Cz-HT and Fl-HT in the presence of 10 vol % boron trifluoride diethyl etherate.

The CVs recorded during polymerisation of In, Cz and Fl with HT in the presence of boron trifluoride diethyl etherate (Figure A4) appear to show rapid electrodeposition. The films obtained have been investigated by IR and Raman spectroscopy, but do not exhibit any signals expected of indole, carbazole or fluorene repeat units (Figure A5 and Figure A6). Similarly, all the films are black and completely insoluble in chloroform. As such, we can conclude that both HT and the other co-monomers are being rapidly degraded during this electrochemical polymerisation experiment, by the combination of applied potentials and the effect of boron trifluoride diethyl etherate.

## 4. Conclusions

Indole (In), carbazole (Cz) and fluorene (Fl) have been successfully polymerised electrochemically and their structures have been confirmed by spectroscopic measurements. In order to improve the solubility of their polymers in organic solvents, we have attempted their electrochemical copolymerisation with 3-hexylthiophene (HT), using a mixture of co-monomers as the solution for polymerisation.

Polymer films have been obtained in all three cases and different co-monomer ratios were employed, yielding thin solid films with slightly differing electrochemical responses. IR and Raman spectroscopic investigations, as well as subsequent solubility testing, revealed that none of the films obtained was composed of linear copolymers.

In the case of In-HT EP and Cz-HT EP films, the respective homopolymers (polyindole and polycarbazole) constituted the films. These homopolymers, however, have been electrochemically substituted at their nitrogen atoms with HT units. Although the length of these HT substituents could not be determined, there was no evidence, either spectroscopic or electrochemical, of the formation of HT polymers or high molecular weight oligomers. As such, we believe that the obtained polyindole and polycarbazole films featured N-substitution by either HT monomers or HT dimers. The HT-grafted polyindole and polycarbazole showed no detectable solubility in chloroform or dichloromethane, which also is indicative of a relatively low share of HT units in their structure.

On the other hand, the Fl-HT EP films showed some solubility in chloroform, leaving only a blackish residue on the electrode. At first, we believed that a soluble copolymer had successfully been prepared. Deposition and spectroscopic investigation of the dissolved species, however, identified it as PHT. As such, instead of a copolymer, a blend of homopolymers was obtained, with the insoluble polyfluorene remaining on the electrode and poly(HT) being dissolved.

We have also attempted to promote copolymerisation, based on the reported method of supplementing the polymerisation solution with BF_3_·Et_2_O. Although the recorded CVs could be interpreted as rapid polymerisation, the films produced showed virtually no electrochemical response and were identified spectroscopically as being mostly over-oxidised and highly degraded, showing more resemblance to a carbon deposit than a conducting polymer film.

## Figures and Tables

**Table 1:** The concentrations of co-monomers in the investigated solutions.

Copolymers/Polymers		Volume Co—Monomers					Concentration [mM]		
	*In*	*Cz*	*Fl*	*HT*	*electrolyte*	*In*	*Cz*	*Fl*	*HT*
In-HT 1-10 EP	0.2 mL	-	-	1 mL	0.8 mL	5	-	-	50
In-HT 5-2 EP	1 mL	-	-	0.2 mL	0.8 mL	25	-	-	10
Cz-HT 1-5 EP	-	0.2 mL	-	1 mL	0.8 mL	-	2	-	10
Cz-HT 5-1 EP	-	1 mL	-	0.2 mL	0.8 mL	-	10	-	2
Fl-HT 1-5 EP	-	-	0.2 mL	1 mL	0.8 mL	-	-	2	10
Fl-HT 5-1 EP	-	-	1 mL	0.2 mL	0.8 mL	-	-	10	2
In EP	2 mL	-	-	-	-	50	-	-	-
Cz EP	-	2 mL	-	-	-	20	-	-	-
HT EP	-	-	-	2 mL	-	-	-	-	100
Fl Ep	-	-	2 mL	-	-	-	-	40	-

In—indole; Cz—carbazole; HT—3-hexylthiophene; Fl—fluorene.

## Appendix A

**Table A1:** Group frequency assignments for major infrared bands observed in obtained materials.

Copolymers/Polymers	Functional Group/Assignments
In; In EP; In-HT EP	ν_In C–H_ 740 cm^−1^; ν_HT C–H_ 992 cm^−1^; ν_BF4-_ 998 cm^−1^; ν_In C–N_ 1197 cm^−1^; ν_In C–N_ 1248 cm^−1^; ν_In C–N–C–C_ 1310 cm^−1^; ν_In N–H_ 1460 cm^−1^; ν_HT C=C_ 1493 cm^−1^; ν_Ar C–C_ 1570 cm^−1^; ν_In Ar C–C_ 1596 cm^−1^; ν_HT C–H_ 2870 cm^−1^; ν_Ar C–H_ 3058 cm^−1^; ν_In N–H_ 3410 cm^−1^
Cz; Cz EP; Cz-HT EP	ν_cz C–H_ 740 cm^−1^; ν_HT C–H_ 810 cm^−1^; ν_Cz C3–C6_ 886 cm^−1^; ν_BF4-_ 998 cm^−1^; ν_Cz C–N_ 1205 cm^−1^; ν_Cz C–N–C_ 1238 cm^−1^; ν_Cz N–H_ 1334 cm^−1^; ν_Cz C–C_ 1397 cm^−1^; ν_HT C=C_ 1450 cm^−1^; ν _HT C=C_ 1500 cm^−1^; ν_In Ar C–C_ 1548 cm^−1^; ν_In Ar C–C_ 1604 cm^−1^; ν_HT C–H_ 2870 cm^−1^; ν_Ar C–H_ 3058 cm^−1^; ν_In N–H_ 3426 cm^−1^
Fl; Fl EP; Fl-HT EP	ν_Fl C–H_ 720 cm^−1^; ν_HT C–H_ 820 cm^−1^; ν_Fl C–H_ 890 cm^−1^; ν_BF4-_ 998 cm^−1^; ν_Fl C–C_ 1185 cm^−1^; ν_Fl C–C_ 1295 cm^−1^; ν_HT C=C_ 1450 cm^−1^; ν_Flz C–H_ 1456 cm^−1^; ν_Fl Ar C–C_ 1600 cm^−1^; ν_Fl Ar C–C_ 1660 cm^−1^; ν_Fl Ar C–C_ 1713 cm^−1^; ν_HT C–H_ 2960 cm^−1^; ν_Ar C–H_ 3040 cm^−1^

In—indole; Cz—carbazole; HT—3-hexylthiophene; Fl—fluorene.
